# Evaluation of a practice team-supported exposure training for patients with panic disorder with or without agoraphobia in primary care - study protocol of a cluster randomised controlled superiority trial

**DOI:** 10.1186/1745-6215-15-112

**Published:** 2014-04-06

**Authors:** Jochen Gensichen, Thomas S Hiller, Jörg Breitbart, Tobias Teismann, Christian Brettschneider, Ulrike Schumacher, Alexander Piwtorak, Hans-Helmut König, Heike Hoyer, Nico Schneider, Mercedes Schelle, Wolfgang Blank, Paul Thiel, Michel Wensing, Jürgen Margraf

**Affiliations:** 1 Institute of General Practice and Family Medicine, Jena University Hospital, Friedrich-Schiller-University, Bachstrasse 18, D-07743 Jena, Germany; 2 Mental Health Research and Treatment Center, Ruhr-University Bochum, Massenbergstrasse 9-13, D-44787 Bochum, Germany; 3 Department of Health Economics and Health Services Research, Hamburg Center for Health Economics, University Medical Center Hamburg-Eppendorf, Martinistrasse 52, D-20246 Hamburg, Germany; 4 Center for Clinical Studies, Jena University Hospital, Salvador-Allende-Platz 27, D-07747 Jena, Germany; 5 Institute of Medical Statistics, Information Sciences and Documentation, Jena University Hospital, Friedrich-Schiller-University, Bachstrasse 18, D-07743 Jena, Germany; 6 Scientific Institute for Quality of Healthcare, Radboud University Medical Centre, Nijmegen, The Netherlands

**Keywords:** Panic disorder, Agoraphobia, Case management, Primary health care, General practice

## Abstract

**Background:**

Panic disorder and agoraphobia are debilitating and frequently comorbid anxiety disorders. A large number of patients with these conditions are treated by general practitioners in primary care. Cognitive behavioural exposure exercises have been shown to be effective in reducing anxiety symptoms. Practice team-based case management can improve clinical outcomes for patients with chronic diseases in primary care. The present study compares a practice team-supported, self-managed exposure programme for patients with panic disorder with or without agoraphobia in small general practices to usual care in terms of clinical efficacy and cost-effectiveness.

**Methods/Design:**

This is a cluster randomised controlled superiority trial with a two-arm parallel group design. General practices represent the units of randomisation. General practitioners recruit adult patients with panic disorder with or without agoraphobia according to the *International Classification of Diseases, version 10* (ICD-10). In the intervention group, patients receive cognitive behaviour therapy-oriented psychoeducation and instructions to self-managed exposure exercises in four manual-based appointments with the general practitioner. A trained health care assistant from the practice team delivers case management and is continuously monitoring symptoms and treatment progress in ten protocol-based telephone contacts with patients. In the control group, patients receive usual care from general practitioners. Outcomes are measured at baseline (T0), at follow-up after six months (T1), and at follow-up after twelve months (T2). The primary outcome is clinical severity of anxiety of patients as measured by the Beck Anxiety Inventory (BAI). To detect a standardised effect size of 0.35 at T1, 222 patients from 37 general practices are included in each group. Secondary outcomes include anxiety-related clinical parameters and health-economic costs.

**Trial registration:**

Current Controlled Trials [http://ISCRTN64669297]

## Background

Based on European epidemiological studies, the 12-month prevalence of panic disorder (PD) is estimated at 1.8% [1]. In 35 to 65% of cases, agoraphobia is comorbid to PD [2]. Typical clinical courses of PD and agoraphobia have been described as chronically recurrent and chronically persistent, respectively [2]. Remissions without treatment were observed in 14% of cases during the course of seven years [3]. Both disorders reveal a disability burden in terms of severe impairments in daily functioning and considerable reductions in quality of life [4]. Compared to individuals without anxiety disorders, patients suffering from panic disorder with or without agoraphobia (PD/AG) show increased health service use and more than three times as many work loss days [5,6]. This results in substantial health-economic costs to society [7,8].

The prevalence of anxiety disorders is higher in routine primary care settings than in the general population [9,10]. Current PD/AG diagnoses were found in about 4% of primary care patients [11,12]. Mental health care for PD/AG is sought and obtained mostly from general practitioners (GPs) [13]. At the same time, these disorders are under-recognised and under-treated in primary care [14,15]. Approximately half of the patients do not receive any anxiety-specific treatment from their GPs [9,16]. Improvements in the management of PD/AG in routine primary care settings have been called for [12,15,17].

### Evidence-based treatments of panic disorder and agoraphobia in primary care

Both psychological and pharmacological interventions are effective in the treatment of PD/AG [18,19]. With regard to psychological interventions, cognitive behavioural therapy (CBT) is considered the ‘gold standard’, since meta-analyses point to its efficacy and effectiveness for all kinds of anxiety disorders [19-21]. In case of PD/AG, research has indicated that CBT is at least equally effective as pharmacotherapy and can result in better long-term effects [22-24].

According to clinical guidelines, primary care therapists can deliver key-elements of CBT to patients with PD/AG as a first step in treatment [25,26]. These key-elements include psychoeducation (offering evidence-based information about disorder and treatment options), bibliotherapy (for example, written self-help books), and motivating patients for self-managed exposure techniques by discussion and instruction. This approach is also known as ‘guided self-help’, where patients independently work through a standardised psychological treatment protocol (a so-called ‘self-help manual’), while receiving additional guidance in terms of few therapist contacts [27]. Meta-analyses found self-help treatments for PD/AG to be similarly effective as traditional CBT face-to-face therapies, since they yield moderate to large effect sizes when compared to non-active control conditions [28-31]. However, in most of the self-help studies to date, guidance on treatment was provided by clinical psychologists or other mental health specialists. Few attempts have been made to determine the efficacy of self-help treatments that were provided by primary care therapists (for example, GPs, health care assistants, nurses). Seekles and colleagues performed a meta-analysis on psychological treatments of anxiety in primary care [30]. They found small effect sizes for treatments that were delivered by therapists who were not specialised in mental health. Hoifodt and colleagues reviewed studies on the effectiveness of CBT that was delivered by primary care therapists for depression and anxiety [32]. Although the authors found that current evidence is limited, they concluded that such treatments are potentially more effective than usual care.

Traditionally, CBT is an amalgam of behavioural and cognitive interventions, including the following elements: psychoeducation, coping skills, cognitive restructuring, exposure exercises (that is to expose oneself sufficiently long and repeatedly to feared stimuli whilst omitting anxiety-reducing avoidance behaviours), and relapse prevention [33]. Out of these elements, exposure exercises are considered essential and therapeutically most important [34]. This view is strengthened by research, showing that solely exposure-based treatments can lead to significant clinical improvements in patients with PD/AG [21,35-38]. Meta-analyses compared the relative effectiveness of the different CBT elements in the treatment of PD/AG [23,39,40]. The findings indicate that teaching coping skills, or using cognitive restructuring, do not increase clinical outcomes above exposure exercises alone (but see the work of Sanchez-Meca *et al*.) [41]. A study by Vögele *et al*. demonstrated beneficial changes in patients’ anxiety-related, dysfunctional cognitions after a solely exposure-based treatment in which no cognitive interventions were used except for explaining the rationale of exercises [38].

### Improving treatment for panic disorder and agoraphobia in primary care by means of a practice team-supported exposure programme

Collaborative chronic care models (CCM) provide an evidence-based framework for improving quality of care for patients with chronic illnesses in outpatient settings [42-44]. CCM are aimed at enabling productive interactions between prepared, pro-active practice-teams and well-informed, motivated patients [45]. Patient self-management support, clinical information systems, and delivery system redesign, by means of stronger involvement of non-physician practice staff in health care delivery, are recommended [43].

Studies indicated that nurse-led ‘case management’ may be a key ingredient of effective collaborative care [46-48]. Case management has been described as a health worker taking responsibility for proactively following-up patients, assessing patient adherence to treatment, monitoring patient progress, taking action when treatment is not successful, and delivering patient support in close coordination with the primary care provider, who retains overall clinical responsibility [46,49,50]. A recent meta-analysis on collaborative care for depression and anxiety concluded that CCM can lead to greater improvements in anxiety outcomes than care as usual [46]. However, only four of the included studies investigated patients with PD, and in all cases study personnel or mental health specialists were involved in patient care [51-54]. As access to specialists is expensive and limited in ‘real world’ primary care settings, it is important to determine the clinical effectiveness and feasibility of case management approaches that can be carried out by practice teams who are not extensively trained in mental health care [55,56].

### Aims and objectives

Clinical effectiveness of primary health care for patients with PD/AG can potentially be improved by guiding patients to CBT-oriented exposure exercises within the framework of practice team-based case management. Focusing on CBT-oriented exposure exercises is clearly most promising in terms of clinical efficacy. It may further enhance feasibility from the viewpoint of practice teams, due to conceptual clarity. Case management procedures seem to be suitable to ensure the required safety of patients as the course of treatment is adequately monitored. If it can be shown that case management can effectively be delivered by health care assistants (HCAs), who are already members of the practice team, possibilities to increase the availability of evidence-based, low-threshold treatments for patients with PD/AG will arise.

The aim of this study is to test the clinical efficacy of a practice team-supported, self-managed exposure programme for patients with PD/AG in small general practices and to evaluate its cost-effectiveness. The study’s primary objective is to determine whether the programme is superior to usual care in terms of lower clinical severity of anxiety at follow-up six months after baseline. Secondary objectives are to determine if the programme is superior to usual care regarding further clinical parameters, patients’ perspectives on receipt of care, and direct and indirect health-economic costs from a societal perspective.

## Methods/design

### Trial design

The study is a cluster randomised, non-blinded superiority trial with two parallel groups. General practices are treated as clusters. Allocation of clusters to two study arms (intervention versus control) is performed with a 1:1 ratio.

Cluster randomisation has been chosen to reduce the chance of contamination of interventions. Blinding is not possible, due to the character of the intervention. However, all patients should be kept blind to the allocation status, until completion of T0 baseline assessment, in order to minimise allocation bias.

### Study setting and eligibility criteria for clusters

The study is conducted in German general practices in which the GP and at least one of his HCAs participate in the study as a ‘practice team’. Inclusion criteria for general practices are: (1) the GP has contracts with all German health insurances (as 90% of care provision is covered by this type of general practices), (2) the HCA has been professionally trained with at least one years’ work experience, (3) GP and HCA give written consent to study-related procedures. An exclusion criterion for general practices is to be specialised for certain diseases or treatments.

### Eligibility criteria for individual participants

Individual participants are patients of participating general practices who have been enroled in the study by their GPs. To be eligible for the trial, patients have to meet the following inclusion criteria: (1) being at least 18 years of age, (2) being diagnosed with PD/AG (ICD-10: F41.0 or F40.01) by a GP-led clinical interview, (3) showing a minimum total score on the ‘Overall Anxiety and Impairment Scale’ (OASIS) of 8 points [57] and at least two positive answers on the panic module of the ‘Patient Health Questionnaire’ (PHQ) [11] at the time of inclusion, (4) having sufficient German language skills, (5) having a private telephone, (5) being capable of giving written informed consent to participate in the study. Patients are excluded if they meet one or more of the following exclusion criteria: suffering from acute suicidal tendencies, acute or chronic psychosis, dependence on psychoactive substance(s), or severe physical illness (limiting life-expectancy to less than one year or limiting feasibility of exposure exercises); being pregnant; receiving professional psychotherapeutic treatment for their anxiety disorder at the time of inclusion. All eligibility criteria for patients must be verified by the GP.

### Interventions pertaining to the cluster level

All practice teams (that is in each case the GP and the HCA of the participating general practice) are initially trained in study procedures and documentation as well as in clinical features of PD/AG. GPs are particularly trained in conducting diagnostic interviews and informed consent discussions with patients. GPs are also trained in current treatment guidelines as usual care should meet recommended standards [26]. Practice teams receive detailed written materials concerning the imparted contents of the training.

All practice teams allocated to the intervention group are additionally trained in treating patients by use of a practice team-supported, self-managed exposure programme. This training comprises the following contents: (1) rationale of exposure techniques with regard to PD/AG, (2) treatment plan, (3) structure of written self-help materials, (4) targeted practice team collaboration with the help of a monitoring checklist, (4a) only for GPs: analysing patients’ feared stimuli, planning and implementing individually appropriate exposure exercises in cooperation with the patient, supervising treatment progress and evaluating success of exposure exercises, possible interactions of exposure exercises with psychopharmacological treatments, (4b) only for HCAs: conducting telephone contacts to patients with the help of a monitoring checklist. Both the GPs and the HCAs receive detailed written treatment manuals.

All trainings should be delivered to the practice teams by educational workshops. In exceptional cases, where attending a workshop is not possible, trainings may be administered by individual Internet-based telephone contacts using Adobe® Connect software (München, Germany). To ensure quality of assessment, documentation and treatment of patients, general practices are contacted bimonthly by members of the project team.

### Interventions pertaining to the individual participants

In the intervention group, the individual treatment plan for patients comprises four manual-based appointments with the GP (about 30 minutes) and ten protocol-based telephone contacts with the HCA (about ten minutes) over a period of 23 weeks. The appointments with the GP are aimed at delivering psychoeducation and instructions to self-managed exposure exercises. The schedule of these appointments is described below. The telephone contacts with the HCA are aimed at monitoring anxiety symptoms and course of treatment approximately biweekly.

Two different kinds of exposure exercises are applied one after another: (1) interoceptive exposure, where patients are exposed to feared bodily sensations and (2) situational (that is *in situ*) exposure, where patients are exposed to feared situations. At the start of treatment, patients receive a newly developed self-help manual that supports all interventions carried out by the practice team. The self-help manual contains psychoeducational information on PD/AG, the treatment rationale, and detailed instructions on self-managed exposure exercises.

The contents and schedule of the manual-based appointments with the GP are as follows: appointment 1 (week 3) - psychoeducation concerning anxiety symptoms, appointment 2 (week 6) - psychoeducation concerning avoidance behaviour and execution of individually tailored interoceptive exposure exercises, appointment 3 (week 12) - reviewing success of interoceptive exposure and planning of individually tailored situational exposure exercises, appointment 4 (week 20) - reviewing success of situational exposure and discussing methods for relapse prevention. Patients are instructed to read the self-help manual continuously and to practice exposure exercises at least twice a week. Patients’ adherence to the treatment protocol is measured by a self-report questionnaire at follow-up T1 (Table 1).

**Table 1:** Outcomes and additional measures

**Measure**	**Description**	**Timeline**			
		**Before baseline**	**T0**	**T1**	**T2**
Primary outcome					
BAI	Clinical severity of anxiety		•	•	•
Secondary outcomes					
MI (subscale ‘alone’)	Agoraphobic avoidance behaviour		•	•	•
PAS (Items A1 and A2)	Number and severity of panic attacks		•	•	•
PHQ-9	Depressiveness		•	•	•
PACIC (short form)	Patients’ perspectives on receipt of care		•	•	•
EQ-5D	Health-related quality of life		•	•	•
CSSRI (modified version)	Health service use and productivity losses		•	•	•
Additional measures					
Screening questionnaire: OASIS; PHQ (panic module)	Patient-reported severity of anxiety symptoms and presence of diagnostic criteria for panic disorder	•			
Diagnostic interview (ICD-10-based checklist)	GP-reported diagnosis of panic disorder and agoraphobia	•			
Comorbid physical and mental disorders	GP-reported ICD-10 diagnoses	•			
Sociodemographic characteristics	Patient-reported age, sex, level of education, and marital status		•		
Treatment adherence questionnaire (self-developed)	Patient-reported adherence to self-managed exposure exercises			•	
GPs’ usual care	GP-reported referrals to psychiatric or psychological treatments (past six months)			•	•

Note. T0 = baseline before intervention; T1 = follow-up at six months; T2 = follow-up at twelve months; BAI = Beck Anxiety Inventory; CSSRI = Client Sociodemographic and Service Receipt Inventory; EQ-5D = EuroQol questionnaire; GP = general practitioner; MI = Mobility Inventory; OASIS = Overall Anxiety Severity and Impairment Scale; PACIC = Patient Assessment of Chronic Illness Care questionnaire; PAS = Panic and Agoraphobia Scale; PHQ = Patient Health Questionnaire. The treatment adherence questionnaire is not administered in the control group.

Patients’ anxiety symptoms and adherence to treatment are monitored by the HCA with the help of a newly developed monitoring checklist (Jena-Anxiety Monitoring List, JAMoL) during ten periodical, approximately biweekly telephone contacts. JAMoL results are reported to the GP. If symptoms worsen or adherence is suboptimal, the GP contacts the patient in order to check for necessary treatment adjustments (for example, changing planned exercises). In case of persistently severe anxiety symptoms or poor adherence to treatment, GPs are recommended to refer the patient to psychiatric or psychological outpatient treatments. However, as the intervention is applied in a real-world setting, GPs retain full clinical responsibility for patients. Therefore, during the whole course of the study, GPs may administer any medical treatments and referrals to any in- or outpatient treatments they deem to be useful.

In the control group, individual patients receive usual care in consideration of recommended treatment standards. GPs retain full clinical responsibility and may administer any medical treatments and referrals to any in- or outpatient treatments they deem to be useful. The contents of usual care during the course of the study are measured at follow-ups T1 and T2 by questionnaire (Table 1).

### Outcomes

The primary outcome parameter is clinical severity of anxiety, as measured by the widely used Beck Anxiety Inventory (BAI) [58,59]. The German version is well-validated. Good to excellent psychometric properties as well as sensitivity to treatment-related changes have been shown in primary care populations [60,61].

Secondary outcome parameters include: agoraphobic avoidance behaviour as measured by the ‘Mobility Inventory’ (MI; subscale ‘alone’) [62,63], number and severity of panic attacks as measured by the ‘Panic and Agoraphobia Scale’ (PAS; Items A1 and A2) [64,65], depressiveness as measured by the ‘Patient Health Questionnaire’ (PHQ-9) [66], patients’ perspectives on receipt of care as measured by the short form of the ‘Patient Assessment of Chronic Illness Care’ questionnaire (PACIC) [67,68], health-related quality of life as measured by the ‘EuroQol’ questionnaire (EQ-5D) [69,70], health service use and productivity losses as measured by a modified version of the ‘Client Sociodemographic and Service Receipt Inventory’ (CSSRI) [71,72], and quality adjusted life years (QALYs) as calculated from the EQ-5D [73].

Primary and secondary outcomes are based on self-report questionnaires that were chosen in consideration of established validity and good psychometric properties in primary care populations. Additional measures are used to address characteristics of the study population, potential confounders of efficacy parameters, or other methodological questions. Table 1 displays the outcome parameters and several of the additional measures. Additional interview studies are planned to highlight the feasibility and acceptability of the intervention from the viewpoint of GPs, HCAs, and patients.

### Recruitment and timeline

General practices are recruited via invitation letters. In a first step, all general practices that are registered by the ‘*Kassenärztliche Vereinigung*’ in Thuringia (Germany) are invited to participate in the study (N = 1,251). If the response rate turns out to be too low to meet the required sample size, general practices from other parts of Germany will be invited by means of public relations. Eligibility criteria for general practices are checked via a questionnaire that has to be filled out by the GP and the HCA. To be enroled, the practice team has to give written consent to follow the study procedures.

Patients are recruited by the participating general practices. The recruitment procedure includes the following steps: (1) Patients of the general practice fill out a screening questionnaire based on the OASIS [74] and the PHQ panic module [11]. (2) Positively screened patients undergo a diagnostic interview conducted by the GP, who thereby uses a diagnostic manual that is based on validated ICD-10 checklists for PD and agoraphobia [75]. (3) If diagnosis of PD (ICD-10: F41.0) or PD/AG (ICD-10: F40.01) is confirmed during the diagnostic interview, other eligibility criteria for individual participants will be checked by the GP. (4) Eligible patients are verbally informed about the study by the GP who also hands out a written information sheet to them.^a^ (5) Eligible patients have to sign an informed consent form to be enroled.

Each general practice is instructed that six patients should be enroled to meet the aspired cluster size. In order to minimise selection bias, it is intended not to randomise a general practice before at least four patients have already been enroled in this practice. Recruitment of these four patients is expected within a time period of eight to twelve weeks. However, if a general practice fails to enrol at least four patients within this time frame, randomisation will be performed anyway.

**Figure 1:**
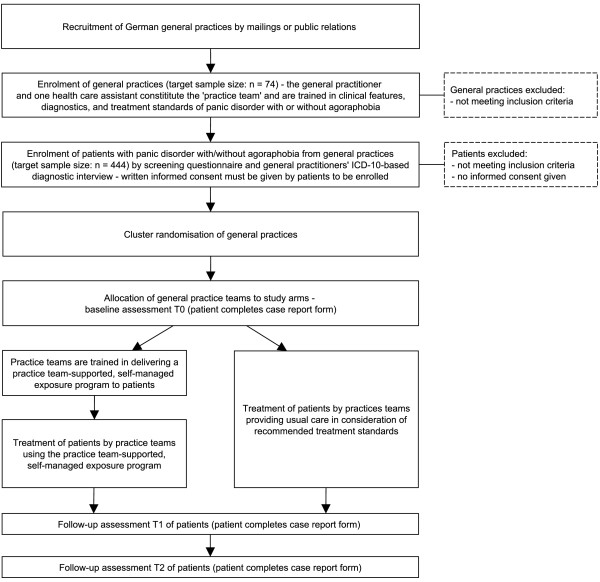
Study flow chart.

Baseline (T0) and follow-up (T1, T2) assessments are administered via self-report questionnaires handed out to patients by the practice teams. Subsequent to T0, the interventions pertaining to individual participants start. Duration of the practice team-supported, self-managed exposure programme is about 23 weeks. Follow-up assessments are six (±one) months after baseline (T1) and twelve (±one) months after baseline (T2). Figure 1 displays the study flowchart.

### Sample size

Main efficacy variable is the sum score of the BAI (values range from 0 to 63). A former study with a German sample of primary care patients observed a standard deviation of 11 and an intra-cluster correlation of 0.07 [76]. Therefore, a standardised effect size of 0.35 (that is 3.85 points on the original scale BAI) would be observed at a significance level of 0.05 by following up 130 patients. However, cluster randomisation is the method of choice for the present study. We expect a cluster size of six patients per practice. In consideration of the resulting design effect of 1.35, and the expected patient dropout-rate of approximately 20%, N = 444 patients from 74 general practices (37 practices per study arm) must be followed-up in order to detect a standardised effect size of 0.35 at a significance level of 0.05.

### Assignment of interventions

Cluster randomisation of general practices is stratified by population density of the administrative district in which the practice is located (that is ‘urban type of general practice’: urban versus rural). Computer-assisted randomisation is prepared by the trial statistician. An uninvolved person, who is not familiar to one of the GPs and not a member of the project team, generates the final randomisation list. The list is provided to the data management via an online randomisation tool.

The assignment of a certain general practice to one of the two study arms is performed by the data management as soon as the general practice is signalling the enrolment of patient(s) via fax. The data management retrieves allocation information for the general practice from the randomisation tool and sends it out to the general practice via fax and phone.

### Data collection and management

General practices collect clinical outcome data by handing out paper-moulded case report forms (CRFs) to patients on schedule (Figure 1). CRFs include patient self-reports on demographic data and outcome parameters (Table 1). As far as possible, CRFs are also handed out to patients who discontinue or deviate from intervention protocols. To ensure quality of assessments, practice teams are extensively trained in data collection procedures, and onsite monitorings are conducted by the project team.

The data management periodically recalls CRFs from general practices. CRFs are visually inspected for missing or ambiguous self-report items. A pre-defined list of self-explaining corrections is applied to CRFs. In cases where self-explaining corrections are not applicable, data queries are conducted by contacting GPs or patients. The paper-moulded CRFs are electronically scanned and then converted to TIF-files using ReadSofts’® FORMS software (Helsingbord, Sweden). Subsequently, the TIF-files are transferred to data sheets using IBM© SPSS® software (IBM Corp., Armonk, NY, USA). Data plausibility checks are performed by the data management. Data are retained for ten years after termination of the trial.

### Statistical analyses

All analyses are based on the intention-to-treat principle, including all enroled participants of randomised general practices providing data under treatment. If necessary, influence of missing data is analysed by means of additional sensitivity analyses.

All population characteristics and outcome efficacy data are presented with adequate descriptive statistics. The primary outcome (total score of BAI at follow-up T1) is analysed by means of a mixed linear model with general practice as random factor and treatment group, baseline value, and urban type of general practice as fixed factors. According to previous research, a normal distribution can be assumed for the present population [60]. The statistical test is performed at a significance level of 0.05 and is regarded as confirmatory. Estimators including 95%-confidence intervals are presented. Secondary outcomes are analysed by mixed linear models or generalised linear models, as appropriate. The tests for secondary outcomes are regarded as exploratory and performed at an uncorrected significance level of 0.05.

During the evaluation of population characteristics, special attention is paid to the success of randomisation. In case of major deviations, additional sensitivity analyses with potential confounding variables are performed (for example, age, sex, type of anxiety disorder under treatment).

With respect to the health-economic evaluation, direct and indirect costs of the treatments in both study arms are calculated. Administrative and market prices are used to valuate health service use. Productivity losses are valuated according to the human capital approach. In terms of an effect measure, quality adjusted life years (QALYs) are calculated from the EQ-5D [73]. A cost-effectiveness analysis is performed from a societal perspective, by calculating incremental costs per QALY. Non-parametric bootstrapping is used to estimate the uncertainty of the incremental cost-effectiveness ratio (ICER). Additionally, a net-monetary benefit regression analysis is performed.

### Patient safety and monitoring of adverse events

The study is planned and conducted in consideration of Good Clinical Practice guidelines (ICH Topic E6, 2002) as well as in accordance with the medical professional codex and the Helsinki Declaration as updated in 2013. Serious risks for patients are not expected as GPs provide continuous medical care to all patients and the interventions under investigation are non-invasive. However, adverse events in terms of unexpected medical problems are monitored and discussed with the studies’ scientific advisory board. The occurrence of any serious adverse events must be reported by the GPs immediately, and decisions about continuation of the study protocol in these individual cases are made. Any decisions concerning the continuation of the whole study are to be made by the principal investigator.

### Research ethics approval

The enrolment of patients did not start unless there was a written and unrestricted positive vote of the local ethics committee. The ethics committee of the Friedrich-Schiller-University at the Medical Faculty (Jena, Germany) approved the study protocol on 17 August 2012 (Approval number 3484-06/12). Protocol modifications are communicated to the ethics committee by amendment.

### Informed consent

Eligible patients are fully informed about the study by their GP and a written patient information sheet is handed out to them prior to their participation. This ensures that patients’ decision about participation is based on knowledge about: anxiety diagnoses and evidence-based therapeutic options; purpose, content, and conduct of the study; potential benefits and risks for their health; data management procedures; and voluntariness of participation. In case of acceptance, patients have to sign an informed consent sheet to be included.^a^ Participants may cancel their participation at any time, without disclosing reasons for their cancellation and without any negative consequences regarding their future medical care.

### Confidentiality of data

All personal information obtained about patients and general practices during the recruitment process (for example, names, addresses, contact details) are held in accordance to the German Federal Data Security Law (BDSG) and medical confidentiality rules. To secure confidentiality, these data are stored in a password-protected server of Jena University Hospital. Paper-based personal information is stored in a locked filing cabinet located at the research office. Access to any personal data is strictly restricted to the project team. Personal information is never passed to any third parties.

Research numbers are assigned to general practices and patients at the time of inclusion. Any clinical data (as obtained by CRFs or other report forms) are encrypted by these research numbers. Clinical data are held strictly apart from personal data and stored on a central server of Jena University Hospital. Encrypted clinical data may be passed to project partners for analysis purposes. In case of individual study cancellations, personal and clinical data are extinguished except that patients explicitly affirm further use.

## Trial status

The trial is ongoing as the project team is still recruiting general practices and patients.

### Endnote

^a^Patient information sheet and informed consent form can be obtained from the authors upon request.

## Abbreviations

BAI: Beck Anxiety Inventory; BDSG: German Federal Data Security Law; CBT: cognitive behavioural therapy; CCM: collaborative chronic care models; CRF: case report form; CSSRI: Client Sociodemographic and Service Receipt Inventory; EQ-5D: EuroQol questionnaire; GP: general practitioner; HCA: health care assistant; ICD-10: *International Classification of Diseases, version 10*; ICER: incremental cost-effectiveness ratio, ICH, International Conference on Harmonisation; MI: Mobility Inventory; OASIS: Overall Anxiety Severity and Impairment Scale; PACIC: Patient Assessment of Chronic Illness Care questionnaire; PAS: Panic and Agoraphobia Scale; PHQ: Patient Health Questionnaire; PD: panic disorder; PD/AG: panic disorder with or without agoraphobia; QALYs: quality adjusted life years.

## Competing interests

The authors declare that they have no competing interests.

## Authors’ contributions

JG: initial conception and design of the study, critical revision and final approval of the manuscript. TSH: conception and design, drafting of treatment manuals, data collection, manuscript writing, final approval of the manuscript. JB: conception and design, data collection, critical revision and final approval of the manuscript. TT: conception and design, critical revision and final approval of the manuscript. CB: conception and design, manuscript writing, final approval of the manuscript. US: conception and design, manuscript writing, final approval of the manuscript. AP: data collection, critical revision and final approval of the manuscript. HHK: conception and design, critical revision and final approval of the manuscript. HH: conception and design, critical revision and final approval of the manuscript. NS: conception and design, critical revision and final approval of the manuscript. MS: data collection, critical revision and final approval of the manuscript. WB: data collection, critical revision and final approval of the manuscript. PT: conception and design, critical revision and final approval of the manuscript. MW: conception and design, critical revision and final approval of the manuscript. JM: conception and design, critical revision and final approval of the manuscript. All authors read and approved the final manuscript.
